# Larger Common River Frogs (*Amietia delalandii*) have Fewer and Shorter Tissue Microplastic Fibres than Smaller Frogs

**DOI:** 10.1007/s00128-024-03852-7

**Published:** 2024-01-28

**Authors:** Mari Burger, Hindrik Bouwman, Louis H. du Preez, Willie Landman

**Affiliations:** 1 Research Unit for Environmental Sciences and Management, North-West University, Potchefstroom, South Africa; 2 South African Institute for Aquatic Biodiversity, Herpetology, Makhanda, South Africa

**Keywords:** Anura, Microplastics, Fibres, Freshwater ecosystems, Tissue concentrations

## Abstract

Little is known about microplastics (MPs) in adult frogs. We investigated MPs in adult Common River Frogs (*Amietia delalandii*) from Potchefstroom, South Africa. Five kinds of samples were analysed: natural water, water used to rinse the skin, skin, intestine, and the remainder of the body (corpus). Tissues were digested. Microplastics occurred in all frogs and sample types (1128 MPs counted). Fibres were the most prevalent MP. Fibre lengths were between 28 and 4300 μm, either polyester or polyvinyl alcohol. MPs in skin were likely derived from the ambient, and MPs in the corpus from translocation via the skin. Fibres in tissues were significantly shorter in larger frogs, a phenomenon we provisionally assign to in situ biodegradation. Microplastics in frogs can potentially be transferred through the food web to higher trophic levels. This study provides the first evidence of MPs in adult frog tissues and avenues for further investigations.

**Graphical Abstract:**

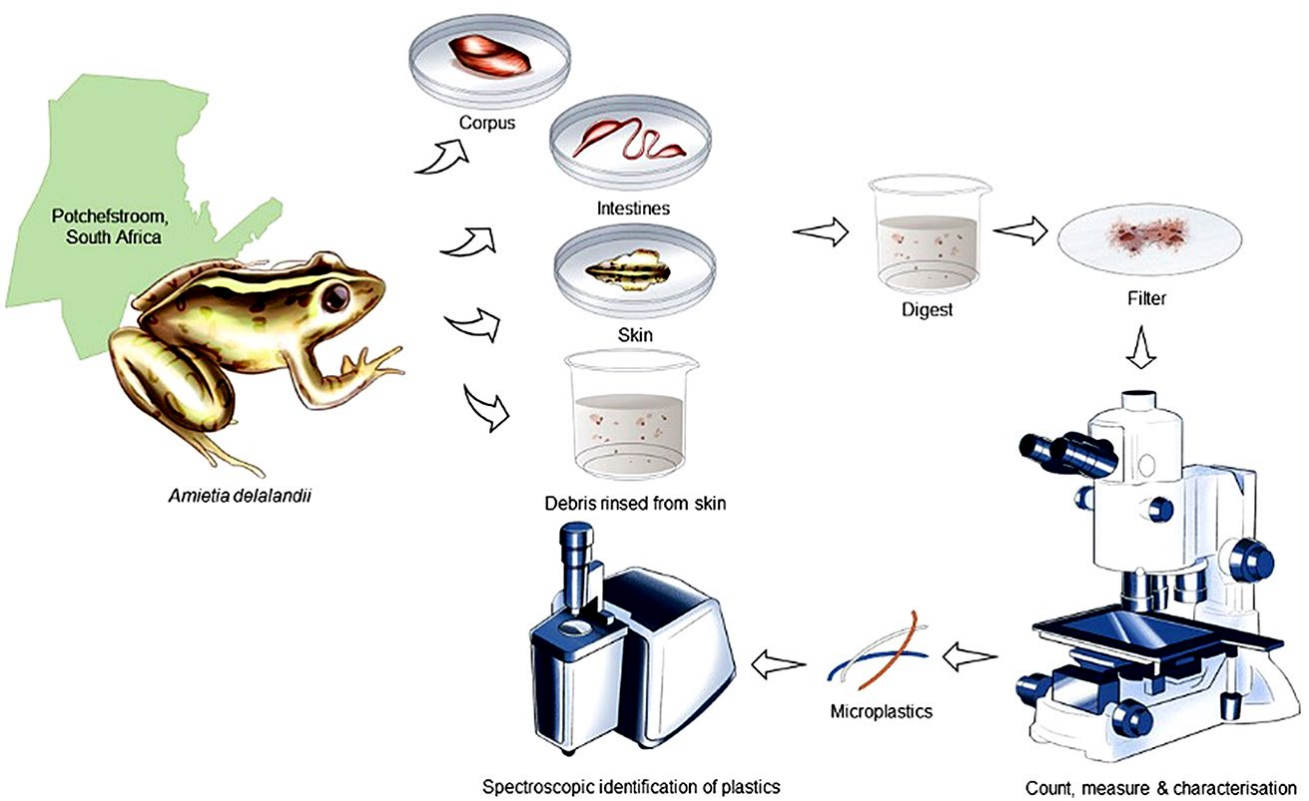

## Introduction

Plastics are synthetic polymers of hydrocarbon molecules mainly derived from natural gas or mineral oil (Crawford and Quinn, 2017). More than 300 million tons of plastic are produced annually, playing intricate and important roles in human society (Rujnić-Sokele and Pilipović 2017). The high demand for plastic is due to its high versatility, low production cost, the ability to produce an immense array of characteristics, and longevity (in most cases) (Silva et al. 2018). Increased production and plastics’ persistent properties, however, make plastic highly abundant and on the increase in the environment (Horton et al. 2017).

Microplastics (MPs) are recognized as any plastic particle smaller than 5 mm in its longest dimension (D’Avignon et al. 2021; Hu et al. 2018; Jiang 2018; Song et al. 2019), and distinguished according to origin. Primary MPs are factory-produced. Secondary MPs are the result of larger plastics degrading into smaller particles (Bonfanti et al. 2021; Song et al. 2019). Plastic degradation can occur through photodegradation, mechanical and biological stress, oxidation, or combinations thereof (Eerkes-Medrano and Thompson 2018; D’Avignon et al. 2021). Microplastics can be further classified as either fibres, fragments, or beads, and sometimes films and foams. Fibres are the most abundant among these and are commonly released from fabrics (Athey & Erdle, 2021; McIlwraith et al. 2019; Napper and Thompson 2016). Primary and secondary MPs, inter alia, can enter rivers and streams through overflow from sewers, farmlands irrigated by wastewater, and runoff from landfills (Yu et al. 2019). Moreover, MPs are easily distributed over wide areas by wind and water due to its small size and low density (Athey & Erdle, 2021; Padervand et al. 2020).

Although MPs have been extensively studied in marine environments, less research is available on freshwater (Kolenda et al. 2020; Verster et al. 2017) and terrestrial environments, although studies here are steadily increasing (Imhof et al. 2012; Horton et al. 2017; Hu et al. 2018; Jiang 2018). These studies suggest that MPs in freshwaters are present in similar or higher quantities compared with marine environments (Imhof et al. 2012; Horton et al. 2017; Hu et al. 2018; Jiang 2018). Due to the ever-decreasing size of MPs, these particles can be ingested by and affect smaller organisms (Silva et al. 2018) inter alia via drinking, feeding, swimming, respiration, adherence to the integument or combinations thereof (D’Avignon et al. 2021). Moreover, incorporated or absorbed chemicals on or in MPs in the body may leach from the MPs into the organism where it may accumulate, and the MPs themselves could from there be transferred to higher trophic levels (Bonfanti et al. 2021; Boyero et al. 2020; Hu et al. 2016).

Amphibians comprise the most threatened vertebrate group globally with 41% of all known species threatened or recently extinct (IUCN 2021). The threats include the loss of natural habitats, emerging infectious diseases, climate change, and pollution (Araújo et al. 2020). Although anurans are vulnerable to these stressors, there is still not much known about MPs on or in adult frogs and how exposure and uptake may affect them. Research concerning frogs and MPs are mainly on tadpoles (e.g., Araújo et al. 2020; Boyero et al. 2020; De Felice et al. 2018; Karaoğlu and Gül 2020; Kolenda et al. 2020). We found only two refereed articles on adult frogs and MPs indicating uptake and possibly accumulation (Pastorino et al. 2022; Tatlı et al. 2022).

**Fig. 1:**
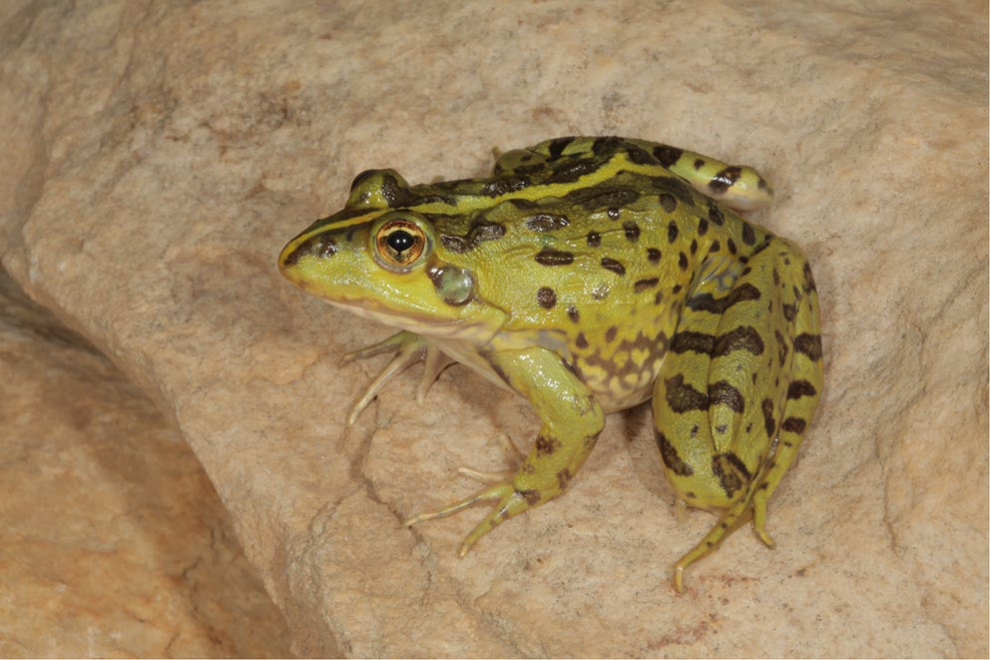
The common river frog *Amietia delalandii* (Photo: Louis du Preez)

The Common River Frog, *Amietia delalandii* (Duméril and Bibron, 1841) (Fig. 1), occurs throughout South Africa, Lesotho, Mozambique, and Zimbabwe in rivers and wetlands (Channing et al. 2016). *A. delalandii* is listed as Least Concern, as it has a wide distribution with a large and stable population (IUCN, 2017). We investigated the occurrence, concentrations, and characteristics of MPs associated with adults of this species, and for the first time to the best of our knowledge, also the occurrence and characteristics of MPs in any frog tissue.

## Materials and Methods

### Sampling

**Fig. 2:**
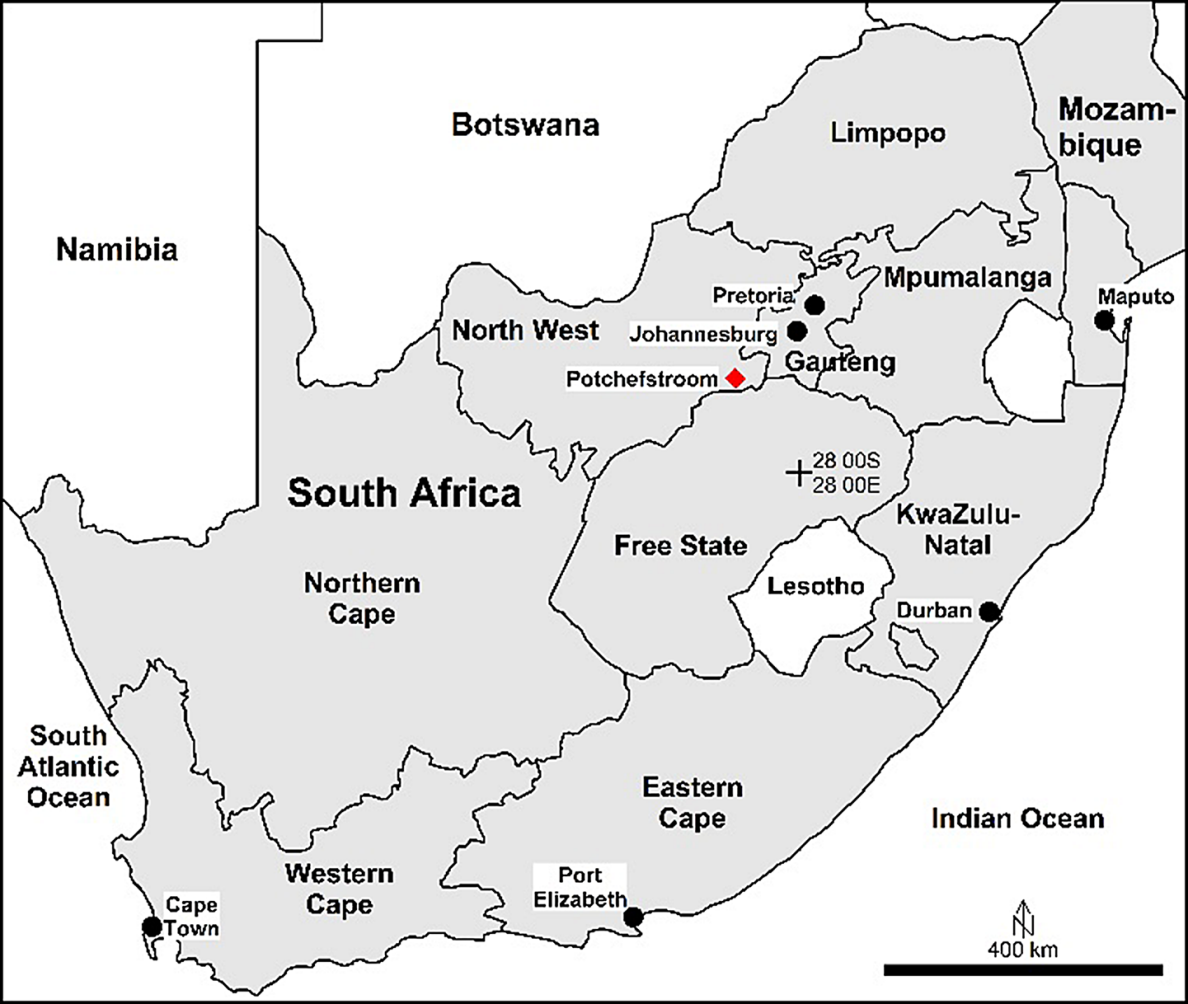
Location of sampling site. Potchefstroom, the collection site, is shown by the red diamond

This project was approved under ethical clearance (NWU-0061-19-A5). To reduce MP contamination during sampling and sample preparation, 100% cotton clothing were worn. Ten *A. delalandii* were collected from a slow-flowing stream (26°39’26.3"S 27°04’46.2"E) on the outskirts of Potchefstroom (26°39’26.3"S; 27°04’46.2"E; South Africa; Fig. 2) by hand, at night, during February 2022. Frogs were euthanised immediately, wrapped in tin foil, placed on ice, and kept frozen until processing and analysis. Twenty-five litres of water, from the stream where frogs were collected, was filtered through a 32 μm sieve and rinsed into a Schott bottle with nanofiltered distilled water, on site. All relevant equipment (tinfoil, Schott bottles, wash-bottles, and sieves) were rinsed, three times, with pre-filtered double-distilled water.

### Processing of Samples and Isolation of Microplastics

Frogs were dissected in a laminar flow cabinet to minimise contamination. Furthermore, precaution was taken to rinse all dissecting tools with prefiltered, double-distilled water before and in between different samples. Ambient MP background, per individual sample preparation, were collected in Petri dishes similar in size to relevant frogs during dissection. All dissections, sample treatments, and MP measurements were done by the first author.

There were five sample types:

1. Water: The 25 L of water, which was collected during sampling, treated as a single reference sample representing the ambient condition.
2. Rinsed skin water: Prior to dissection with the frog still whole, all debris and MPs adhering to the external of the frog was rinsed with 10 ml, double-distilled, pre-filtered water. It is considered part of the frog in terms of trophic transfer considerations, but not part of the skin tissue—therefore representing non-tissue incorporated MPs.
3. Skin: After rinsing, the whole skin was removed and digested to count and characterise MPs in the skin tissue—therefore representing tissue-incorporated MPs.
4. Intestine: The complete intestine was dissected and digested. The intestine was not cleaned beforehand, so the concentrations and characterizations represent intestine content and MPs in the intestinal tissue. Since we could not distinguish between tissue-incorporated and non-tissue incorporated MPs (most likely a mixture), we considered this sample type as non-tissue incorporated.
5. Corpus: The remainder of the carcass, sans skin and intestine, was treated as a complete sample to count and characterise the MPs in the rest of the body tissues—therefore representing tissue-incorporated MPs.

Tissue samples were weighed individually, frozen in covered glass Petri dishes, and thawed prior to digestion. Water and tissue samples were digested in a 2 M NaOH (at 10 ml/g sample) and 0.5% sodium dodecyl sulfate (SDS), (at 5 ml/g sample) solution (adapted from Ferreira et al. 2022) to eliminate non-plastic organic material. Samples that weighed less than one gram were digested in 20 ml NaOH and 10 ml SDS. Samples were digested at 50 °C for 24 h while being magnetically stirred in a fume hood. The digestates were filtered through custom-made 25 μm stainless steel sieves with vacuum to isolate MPs. Filters with MPs were dried individually overnight in covered glass Petri dishes. A method recovery test was conducted, whereby 20 polyester fibres were put through the same digestion method to determine recovery efficiency.

### Characterisation of Microplastics

Microplastics were counted, measured, and characterised using a Nikon AZ100M microscope (Nikon, Tokyo, Japan) with a 1 × objective. Images were taken with a Nikon Digital Sight DS-Fi2 (Nikon, Tokyo, Japan) digital camera. Microplastics morphology was categorised as either fibres, fragments, or beads (regular spherical shape). All MPs were counted, measured to maximum length, and the colour noted by eye.

Confirmation of MP polymers was with attenuated total reflection (ATR) infrared analysis with two MPs. The others were too small to fit. Spectra obtained was compared to a reference FTIR spectrum (S.T. Japan-Europe GmbH MP). Data was collected at a resolution of 8 cm^− 1^ with 32 scans per sample. Ambient background MPs were subtracted per MP category and colour. For instance, if the background had three white fibres, then three white fibres were subtracted. Concentrations per sample (skin, skin water, intestine, and corpus) were calculated and expressed as n/g (numbers per gram wet tissue). In most cases, values were rounded to two significant numbers and reported as number per gram (n/g). Skin water concentration was calculated on the number of MPs in the 10 ml of water used for rinsing divided by the mass of each skin sample and expressed as number per gram skin (n/g skin).

### Data Analysis

Statistical analysis was performed using GraphPad Prism version 10.0.0. Data were log-transformed. One-way ANOVA was followed by Tuckey’s post-hoc tests for MP concentrations and fibre lengths between sample types. To test for differences in proportional fibre colour compositions between sample types, a chi-square test was performed on counts with colours classified as white, red, blue, and other. Linear regressions were performed to test whether there was a significant association between concentration of fibres and sample mass, and between fibre length (all fibre lengths in skin, intestine, and corpus combined) and frog mass.

## Results

### Extraction

**Fig. 3:**
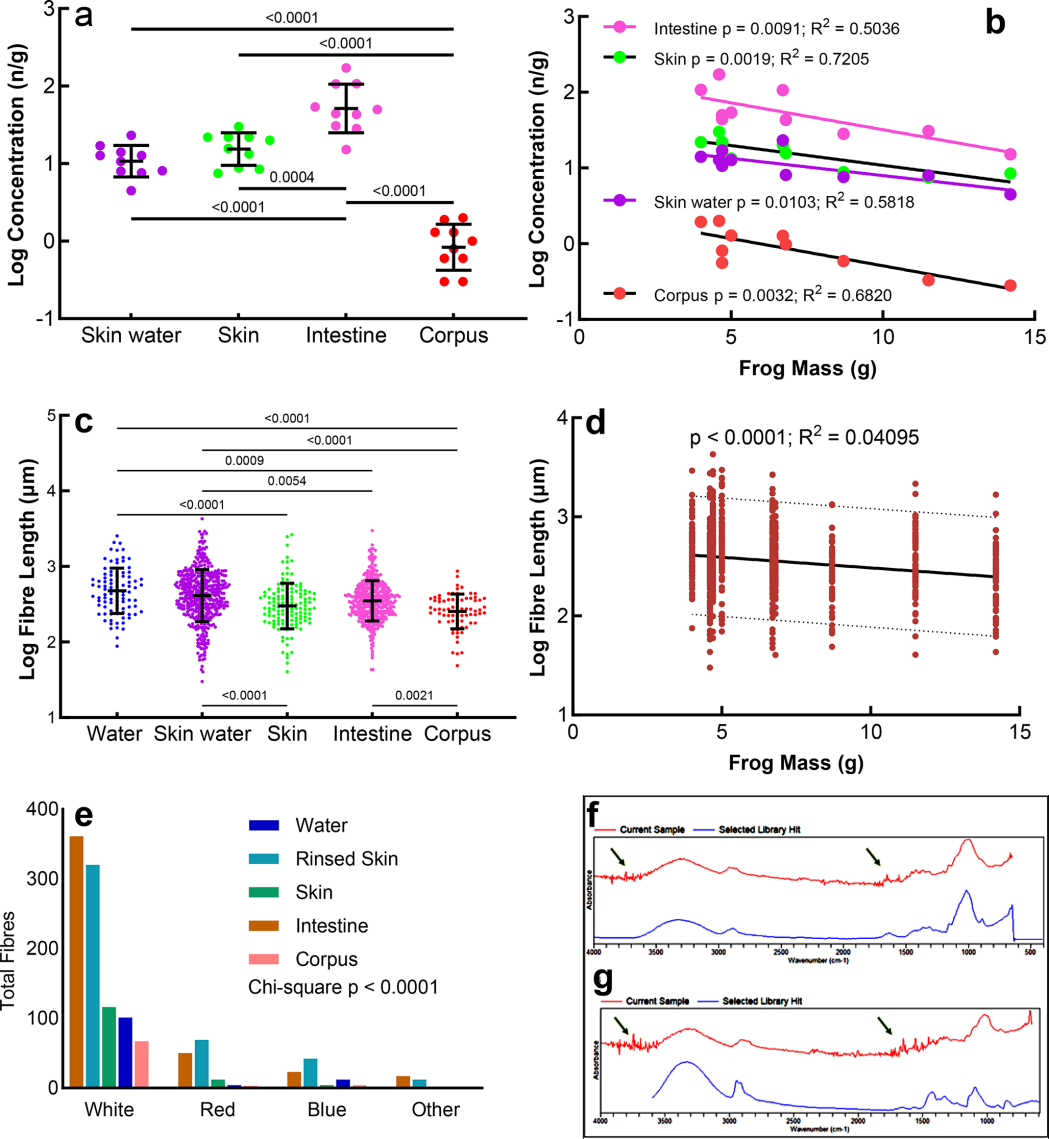
Scatterplots of ANOVAs between sample types (**a** and **c**), linear regressions (**b** and **d**), and chi-square analyses of colour compositions (**e**). Dotted lines on Fig. 3d are 95% confidence intervals. Analysis with FTIR, spectra of two microplastic samples (**f** and **g**). Analysed fibre samples are shown in red with the reference spectra in blue. Green arrows indicate water vapour absorption. (**f**) Polyester fibre with a 92% match. (**g**) Polyvinyl (alcohol) with a 92% match

Eighteen of the 20 white fibres used for the recovery test were found indicating a good extraction and recovery procedure. Beads were not found imbedded in the skin, nor were there any in the corpus. Fragments were also not present in the corpus. Microplastics were found in all sample types, 98% of which were fibres. We could only characterise MPs found in skin tissue as either incorporated in the tissue or recalcitrant to rinsing. However, we consider these MPs as tissue-incorporated as their mean fibre lengths were significantly shorter compared with rinsed skin fibre lengths (390 μm in skin vs. Figure 3c).

### Frogs and Microplastics

**Fig. 4:**
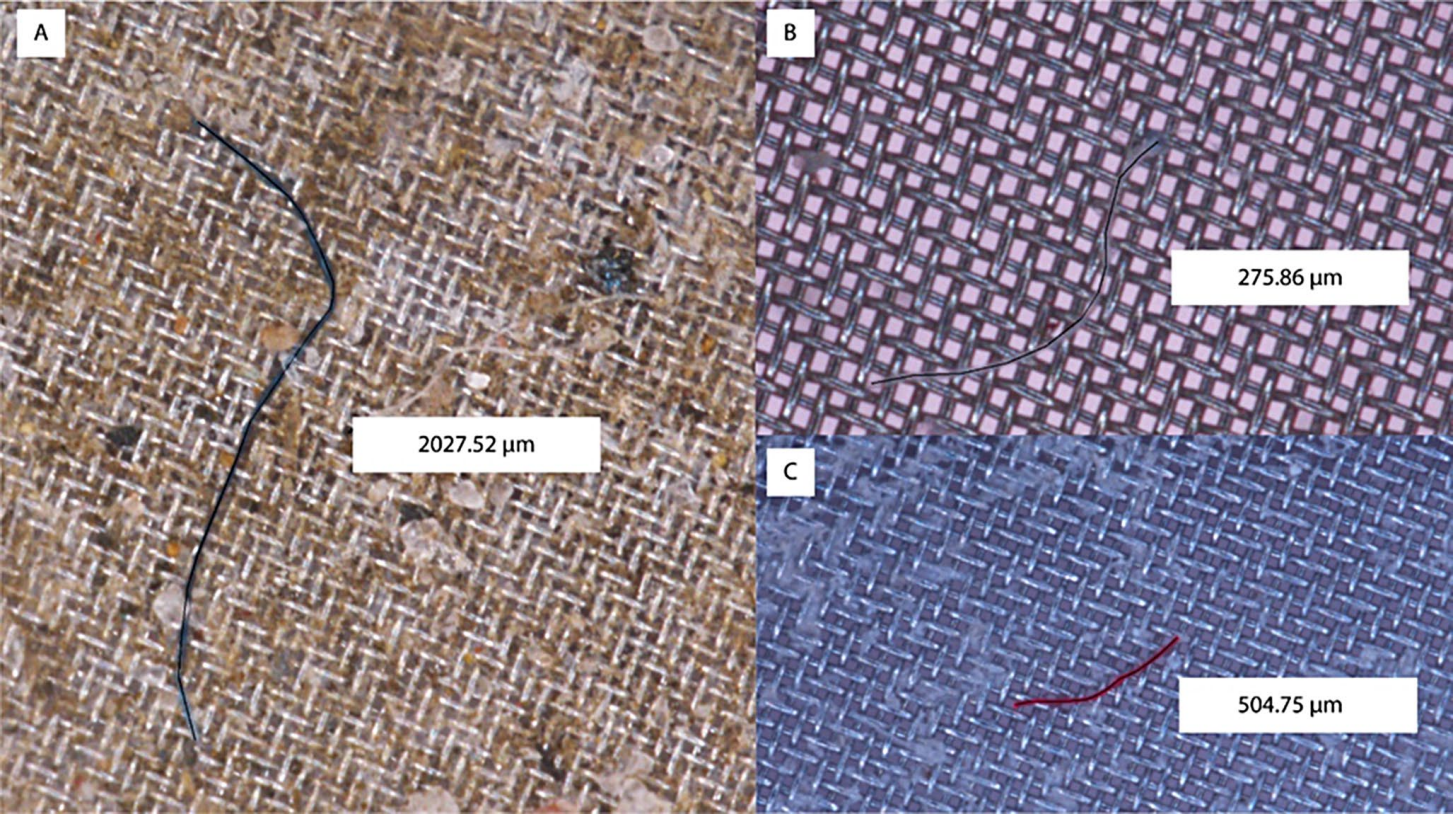
Examples of coloured fibres and their corresponding lengths. (**A**) Blue fibre. (**B**) White fibre. (**C**) Red fibre

The ten frogs had a mean mass of 7.1 g with a minimum of 4.0 g, and a maximum of 14 g. A total of 1128 MPs were isolated and counted (Fig. 4). Microplastics occurred in all frogs and all sample type samples (Fig. 3; Table 1). Of the 1128 MPs, ten were beads and 17 were fragments (Table 1). Beads and fragments were not included in subsequent analyses.

**Table 1:** Concentrations (n/g wet mass) of microplastics in sample types

	Water*	Skin water	Skin	Intestine	Corpus
N frogs		10	10	10	10
Minimum		14	7.5	15	0.30
Maximum		126	30	172	2
Mean		69	17	65	1.0
Median		71	18	47	0.90
Geometric mean		57	15	52	0.84
SD		39	7.4	49	0.61
%CV		56	44	75	80
Beads (n)	0	1	0	9	0
Fragments (n)	1	9	2	5	0

*Concentration in ambient water could not be calculated

### Concentrations

Since we only characterised an unknown proportion of the MPs filtered from the ambient water sample, we could not calculate a confident MP concentration (Table 1). The corpus samples had the lowest mean concentration at 1.0 n/g. Skin, the only other complete tissue type, had a mean of 17 n/g, while intestines (with contents) contained a mean of 65 n/g. These two tissue types had the highest %CV. The Anova showed that all concentration medians differed significantly from each other, except between skin water and skin (Fig. 3a). All concentrations in tissues also declined significantly with frog mass (Fig. 3b). Comparisons of the slopes revealed no differences (*p* = 0.5729)—concentrations of MPs in all tissues therefore decreased with frog mass at the same rate.

### Fibre Lengths

Fibres in the ambient water were the longest at a mean of 600 μm, while the fibres in the corpus were the shortest at 290 μm (Table 2). The longest fibre was 4300 μm in skin water, with the longest fibre in the corpus at 900 μm. The median fibre lengths differed significantly between all sample types, except, again, between ambient water and skin water (Fig. 3c). Per sample type, the concentrations of fibres decreased significantly with increased frog mass (linear regressions; Fig. 3b). Per frog, the fibre lengths (all fibres in skin, intestine, and corpus combined) became significantly shorter (*p* < 0.0001) with increased frog mass, although the R^2^ was low, at 0.04 (Fig. 3d).

**Table 2:** Mean fibre lengths (µm) per sample type

	Water	Skin water	Skin	Intestine	Corpus
n	95	443	133	450	75
Minimum	88	30	41	43	49
Maximum	2500	4300	2600	3000	900
Range	2400	4300	2600	2900	820
Mean	600	550	390	430	290
Median	470	440	290	340	270
Geometric mean	480	410	300	350	250
SD	460	480	380	320	140
%CV	76	87	96	74	50

### Fibre Characteristics

Of all the fibres combined, white fibres made up 79.1%, red made up 11%, blue was at 7.0%, and other colours combined were 2.6%. The chi-square analysis of colour proportions between sample types showed a probability of less than 0.0001 that the differences in proportions can be ascribed to chance (Fig. 3e). The colour proportions are therefore significantly different between sample types.

Microplastic analysis using an ATR-FTIR had limited success (Fig. 3f and g). Small MP fibres yielded insufficient spectrum intensities under high resolution (8 cm^− 1^) and number of scans (32 scans per sample). Furthermore, water vapour absorption from the atmosphere further reduced the quality of already weak spectra, making it hard to determine polymer types (Hammerli et al., 2021). Only 18 fibres were large enough to be removed by forceps for ATR-FTIR analysis. Thirteen of 18 particles analysed were unidentifiable due to an insufficient spectrum intensity. We found polyester and polyvinyl fibres (Fig. 3f and g) but could not determine proportional polymer compositions.

## Discussion

### Concentrations

Eighteen of the 20 white fibres used for the recovery test were found indicating a good extraction and recovery procedure. Beads were not found imbedded in the skin, nor were there any in the corpus. Fragments were also not present in the corpus, suggesting that neither beads nor fragments were translocated to internal tissues. Microplastics were found in all sample types, 98% of which were fibres. We could only characterise MPs in skin tissue as either incorporated in the tissue or recalcitrant to rinsing. However, we consider these MPs as tissue-incorporated as their mean fibre lengths were significantly shorter compared with rinsed skin fibre lengths (390 μm in skin vs. Figure 3c). This becomes important in later discussion.

Rinsed skin water had five times more MPs than the MPs incorporated in the skin tissue itself (69 and 17 n/g, respectively), and the difference was significant (Table 1; Fig. 3a). MPs adhere therefore to frog skin despite the frog being caught and handled by hand. Since there was no difference in mean fibre lengths between ambient water and rinsed skin water (600 μm and 550 μm, respectively; Table 2), it can be assumed that the fibres in the skin were incorporated directly from the ambient environment and not via ingestion and subsequent translocation. The fibres in the intestine and corpus were also shorter than skin fibres (Table 2; Fig. 3c), an argument against translocation of MPs to the skin via ingestion.

Frog skin has specialised adaptations which perform a variety of physiological functions (water uptake, respiration, etc.), while still being able to maintain a selective barrier to the surrounding environment (Liewelyn et al. 2019; Varga et al. 2019). It should be noted that experimental studies found that the primary uptake of water by adult frogs (*Rana pipiens* (now, *Lithobates pipiens* Schreber, 1782), *Bufo marinus* (now, *Rhinella marina* Linnaeus,1758), and *Xenopus laevis* Daudin, 1802) is via the skin (80–90%) and the rest by secondary oral uptake, but not active drinking (Bentley and Yorio 1979). In *L. pipiens* (the closest relative to *A. delalandii* studied by Bentley and Yorio (1979), the oral uptake of water was between 1.4 and 5.4% depending on the hydration treatment. We deduce that MP uptake for *A. delalandii* in the present study was mainly via skin.

Individual MPs in skin tissue may be transient due to depletion by sloughing, replaced by chronic contact to MPs with the immediate environment. It would be instructive to subsample functional parts of the skin. The softer belly skin might experience higher contact with MPs in sediments and soils, while the head and snout skin may have lower concentrations but shorter fibres due to friction or abrasion with objects in the environment during movement.

MP concentrations in skin water and intestine were almost the same suggesting that the majority comes from ambient water (Table 1; Fig. 3e). However, ambient water had significantly longer fibres than any other sample type except skin water, suggesting selective uptake via ingestion. It is possible that the small prey of the frogs also contains relatively shorter fibres, explaining the shorter fibre lengths in the intestine. Although Jâms et al. (2020) concluded that larger animals ingest larger MPs, this was a large-scale assessment. They found considerable variation but amphibians were not included.

Complicating these seemingly obvious explanations are the regressions in Fig. 3b and c. Concentrations and fibre lengths in frogs decrease and become shorter with an increase in frog mass for any sample type (all linear regressions *p* < 0.02). Dilution by growth could be a partial explanation if the majority of the tissue-incorporated MPs were taken up in pre-metamorphosis. The plasticity of plastic fibres is such that any differential pressure that might cause breakage inside tissues is unlikely, and if it did occur, would have resulted in increased fibre concentrations, not a significant decrease as we found. Also, the fibre lengths in skin water and intestine could be assumed to reflect the ambient and not age-related factors, but even here, larger frogs had shorter fibres. Larger frogs likely eat larger prey, possibly with more and longer fibres (Jâms et al. 2020) but this is countered by the regressions. Frogs seldom masticate their prey which may cause shortening of fibres. On the other hand, Antarctic krill *(Euphausia superba*) for instance, produce smaller plastics in the digestive gland (Dawson et al. 2018). It could also be that larger frogs differentially eliminate longer fibres with age which may explain the differences in colour compositions between sample types (Fig. 3e), lower concentrations, and shorter fibres.

However, there may be another approach to this conundrum, namely in-organ biodegradation of MPs. To the best of knowledge, no one has studied the biodegradation of microplastics as a pollutant within tissues. There is a body of work though, on biomedical implants that considers the ‘fatigue’ and biodegradation of materials such as metals, ceramics, and polymers implanted for medical and research reasons. Polymeric implants suffer degradation in vivo and in vitro via hydrolysis and oxidation, depending on polymer composition, reactivity, elasticity, and morphology, among a host of other factors (Acemoglu 2004). The degradation takes the form of surface cracking and pitting, with release of corrosion products, additives, and contaminants (Acemoglu 2004; del Prever et al. 1996; Williams 2008). It therefore can be deduced that a range of fibres of different polymeric compositions will biodegrade at different rates with more susceptible polymers becoming shorter quicker. Indeed, fibres may become too short to be detected by the detection method used, possibly explaining the lower concentrations we found with age. If in situ biodegradation were the case, one would expect the fibres to become thinner as well; something that we did not measure but should be considered in the future.

Taken all together, we have no integrated explanation encompassing all our findings regarding allometric relationships, except that a number of factors may interact differently with age and somatic development.

### Comparisons with Published Data

Comparison with MPs in adult frogs was difficult. Pastorino et al. (2022) captured five *Rana temporaria* in northern Italy and analysed their intestines through enzymatic digestion. Concentrations were not reported. They reported only one fibre per frog intestine, the fibres were between 0.5 and 2 cm long. In contrast with our findings, larger frogs had longer fibre lengths. Differences in analytical processes and local background MP concentrations might explain the differences between our studies.

Tatlı et al. (2022) did not report concentrations in the intestine of *Pelophylax ridibundus* captured in various places in Turkey, finding 1 215 MPs in 147 of the176 frogs they captured, at a mean of 8.3 MPs per positive frog. We found 450 fibres in ten frog intestines at a mean concentration of 45 per frog (or 65 n/g; Tables 1 and 2), with a mean length of 430 μm (Table 2). Tatlı et al. (2022) did not measure lengths and found no association between numbers in the intestine and frog length or mass.

Fibres dominated as the main MP type we found as did others reporting on frogs (Pastorino et al. 2022; Tatlı et al. 2022). White fibres were the most prevalent in the present contrast to other studies on anurans, which found blue/navy/black to be the most prevalent colour (Kolenda et al. 2020; Pastorino et al. 2022; Tatlı et al. 2022). Polymer types detected in this study were either polyester or polyvinyl (alcohol) (PVA). This agrees with Hu et al. (2018), where polyester fibres were the most prevalent polymer type found in tadpoles.

The variations and differences of the little data available probably reflect a combination of different analytical methods, frog behaviour and biology, and environmental background. This does mean though that the assessment of risks posed by MPs to anurans remains poorly understood.

### Effects

Microplastics have been shown to alter the feeding efficiency of tadpoles, either by inducing early satiety, (Balestrieri et al. 2022) or by causing direct damage, affecting the tadpoles’ growth and survival (Araújo et al. 2020). MPs have the potential to bioaccumulate as the tadpoles mature. Bioaccumulation of MPs in the intestinal wall can induce mechanical stress (Bonfanti et al. 2021) or immunosuppression causing an increased vulnerability to pathogens (Kataoka and Kashiwada 2021). False satiety caused by MPs in the gut has similarly been seen in various animals including earthworms (Zhang et al. 2022), lobsters (Welden and Cowie 2016), and turtles (Santos et al. 2020). This causes reduced feeding intake, leading to weight loss and nutrient deficiencies, which negatively influences the fitness of animals and their populations (Santos et al. 2020). Additionally, MP size affects the length of time an item remains in the organism; gut retention time of MPs increases with MP decreasing size (Yu et al. 2021; Fernández and Albentosa 2019). The increased false satiety in organisms may lead to a decrease in fitness. This is critical in frogs as they are already threatened globally, with MP pollution adding more stress to their populations. It should also be kept in mind though, that research on wild animals is done on survivors of all the combination of stressors, and that badly affected individuals would not likely be captured or have already died.

### Synthesis and Recommendations

Frogs are important links in the energy flow in trophic freshwater systems (Oliveira et al. 2021) as both predator and prey. Moreover, there is a transfer of MPs between freshwater systems and frogs as the amphibious and larval nature of frogs suggest that MPs from the water environment may return to terrestrial environments, from whence the MPs originally came. Uptake of MPs can occur directly from the environment or indirectly through the consumption of aquatic and terrestrial prey that make up adult frogs’ diet (Tatlı et al. 2022), but not via drinking (Bentley and Yorio 1979). Microplastic fibres for instance can enter the body of fish through respiration (Li et al. 2021). This is probably true for anurans as well, especially during the tadpole stages. Furthermore, anuran tadpoles are known to actively feed on MPs (Balestrieri et al. 2022).

We found MPs in all sample types in adult *Amietia delalandii* from a stream in South Africa. Fibres in frog tissue seem to be taken up and incorporated in different ways in skin and corpus tissues. Future studies on MPs need to bridge the gaps in research regarding exposure, uptake and the mechanisms facilitating potential harmful effects on adult frogs, biodegradation in tissues, the possibility of trophic transfer, and the consequences for ecosystem functioning. Further studies need to be conducted on MPs affecting the feeding efficiency in adult frogs. Moreover, research needs to address the occurrence of MPs in different species of frogs associated with aquatic and terrestrial habitats, to understand the potential threats to their populations. Our study provides novel insights into the occurrence and characteristics of MPs in adult anurans. We present the first data of MPs in adult frog tissues and uncovered poorly understood allometric phenomena such as lesser fibre concentrations associated with shorter fibres in larger frogs that may be ascribed to biodegradation in situ. We also compared colour distributions between frog-associated matrixes for the first time and found significant compositional differences we could not explain. Our findings are relevant regarding the increasing concern about MPs in fresh waters combined with the decline of amphibians globally. Our observations will inform amphibian risk assessments and provide relevant targets for further research.
